# On Some Cases of Congenital Fissures of the Mouth

**Published:** 1888-04

**Authors:** J. Bland Sutton


ARTICLE IV.

### ON SOME CASES OF CONGENITAL FISSURES OF THE MOUTH.

BY J. BLAND SUTTON, F. R. C. S.,

In 1867, Sir William Fergusson, in his well-known lecture on Hare-lip and Split Palate, states "that hare-lip in the human subject bears no resemblance to that of the hare. In this animal it is invariable in the middle line; in man it never is." Among the many cases which this surgeon saw, none were in the middle line.

Mr. Holmes, commenting on this statement, amends it thus:—

"The natural cleft in the lip of the hare differs from the unnatural cleft in the human lip in the important particular of being in the middle line, which the human hare-lip never is, or so rarely that it may practically be said to exist."

It is also equally noteworthy that in hare-lip occuring in mammals the cleft is commonly situated to the right or left of the median line.

A similar condition is present in the lip of the lamb presented to the Museum of the Odontological Society by Mr. Willoughby Weiss. In this specimen the fissure is on the right side. Although one would imagine hair-lip to be fairly frequent in the lower mammals, to judge from its prevalence in the human kind, nevertheless the two cases just mentioned are the only actual specimens I can refer to at the present moment, and no other recorded cases are known to me.

Median hare-lip, however, occurs in the human subject, and the specimen, for which I am deeply indebted to the courtesy and kindness of my friend, Mr. Frederick Treves, establishes this beyond all doubt.

On examining the child before its death, I felt convinced that there was no ethmo-vomerine plate, and this conviction was strengthened by the peculiar shape of its forehead. When the child died, this opinion was fully confirmed; there was no ethmo-vomerine plate, consequently no nasal septum, and, what is more important, the premaxillary bones were absent. The bearing of this peculiarity will be recognised when we come to discuss the embryological features of these fissures.

In the summer of 1866, whilst staying in Paris with my friend, Mr. W. H. Freeman, of Bath, we noticed some pug dogs, kept as fancy pets, with remarkable clefts in the upper lip and nose. A bitch was purchased, and at Bath Mr. Freeman had her crossed with a Skye-terrier. The result was successful; half the pups presented well-formed lips and noses, the other half had cleft noses like the mother.

The deformity consists of a median vertical split involving the upper lip, extending some distance between the nostrils, and passing between the incisive bone, opens on the hard palate.

The defect is of great interest; for median hare lip is excessively rare, and even in the hare the mesial cleft is confined to the lip, and does not involve the nose. It is also interesting in that it serves as a good example of a defect being transmitted until it becomes an established condition. It is very difficult to explain, on the ordinarily received opinion regarding the development of the nose and upper lip, how this defect comes about. An examination of the lip of a normal dog, however, shows that the cleft, or cicatrix, present in the dog's upper lip, extends some distance along the nasal septum. Hence it is possible that we have in this case to deal with an arrest of the coalescence which normally takes place in this situation.

Equally true is median fissure of the lower lip, not only in man, but in mammals. Sir William Fergusson figured an example of this defect, the only one he ever saw in his exceptional experience of these cases.

An excellant example of median fissure in the lower lip, involving the symphisis and tongue of a calf, has been placed on record by Dr. Joseph Walker. A very remarkable specimen is recorded by Lannelongue. It was observed in a child two years and a-half old. The split involved the lower lip, and between its edges a tumour existed. This was removed, and the edges brought together. The lower incisors were natural.

Judging from the figure accompanying Lannelongue's account of the case, it would seem to be a dermoid, and to that variety to which I have applied the term sequestration cyst.

Before proceeding to discuss some points connected with the development of the parts under consideration, we must study yet another example of abnormal fissure, viz., *macrostoma*. This congenital defect is due to failure of union, partial or complete, of the mandibular fissure beyond the natural limits of the mouth.

Through the courtesy of Dr. Rayner and the kindness of my friend, Mr. John Palmer, I have been able to study and exhibit a remarkable specimen of this rare defect.

In this instance the child, when born, was found to possess an unusually large mouth, the angles of which gradually passed into a red cicatrix. This scar in its turn ended in a gasping recent wound over the temporal region extending over to the dura mater. The condition was symmetrical.

The impression left on the mind of those who saw the case was, that the injury had been caused by a tight amniotic band engaging the mouth at the time of birth and impending delivery, especially as the nurse's attention had been arrested by a strong cord-like piece of amnion. This opinion, however, connot be entertained.

Recently, through the kindness of Mr. G. Seymour, an opportunity has been afforded me of studying a very interesting instance of what may be considered the mildest form of the defect. The patient, a little girl aged seven years, presented on the right side of the cheek, an inch and a-half behind the angle of the mouth, a small depression surmounted by a tiny cutaneous nodule. The depression in the skin was about one-sixteenth of an inch deep. Corresponding with this, on the mucous membrane of the mouth, was a white cicatrix one-fourth of an inch in diameter.

On the left cheek, an inch and a-half behind the angle of the mouth, is a small congenital cutaneous elevation. The pinna on this side is very defective, and as far as my examination extended, the external auditory meatus is covered with skin; but I do not think the meatus is completely occluded, because the girl has some hearing on that side.

The case is one of extreme interest, and may be interpreted as follows:—

The dimple on the right cheek and the nodule in the left one result from the faulty closure of the edges of the mandibular cleft, in the same way that imperfect coalescence of the branchial fissures leads to the formation of cervical branchial fistulæ.

Macrostoma is, as has already been mentioned, frequently, but by no means always, associated with defects of the auricle. It is of great interest to find in this case the defects in the line of the mandibular cleft associated with an abnormal auricle.

As far as my knowledge of the literature of this subject extends, this is the only recorded case of a congenital depression in the cheek allied to branchial fistulæ.

Some amount of new light appears to have been shed on this matter by His's careful investigations into the anatomy of early human embryo. This writer's account is somewhat after this fashion:—The mouth in a human fœtus of the fifth week is represented by an opening from which five fissures radiate. The upper pair are the orbito-nasal, the two lower form the mouth, whilst the median fissure separates the lower jaws. As the median process develops to form the nose, two rounded prominences make their appearance at each angle. These may be referred to as the globular processes. From the globular processes the alæ of the nose and intermaxillæ are derived; later they are joined by the lateral pieces to complete the lip.

He further points out that in some mammals, especially the rodents, the globular processes do not fuse together, but are permanently separated; whereas in man they always fuse together in the middle line, but are not so constantly joined by the lateral pieces.

These facts are of value, inasmuch as they afford a ready explanation of the occurrence of median hare-lip in the lower mammals, but its extreme rarity in man. When it occurs in man it is due to an arrest of development of the globular processes. Hence we may fairly infer that, in the variety of pugs already referred to, the globular processes habitually fail to coalesce in the median line. It is open to question whether the globular processes owe their origin entirely to the fronto-nasal plate. The case of median fissure, which has been mentioned, would seem to show that the premaxillæ arise in connection with the ethmo-vomerine plate, and that the skin covering them is derived from the fronto-nasal plate; for in that specimen the alæ nasi are well-formed, but the premaxillæ and septum are absent.

Hare-lip in man is not necessarily accompanied by a split palate, but if cleft palate is associated with hare-lip, the cleft involves the entire palate. If His's view concerning the globular processes is correct, then it would be expected that hare-lip could be associated with a cleft involving only the premaxillary bones.

Whilst studying these abnormal fissures about the mouth, a marsupia lembryo (*Macropus*), which could only have been in the pouch two or three weeks, came under my notice, and the condition of its mouth is so interesting that I have had it drawn. It presents a median cleft in the upper lip, which differs from the hare's cleft in that it involves the premaxillæ exactly as in the case of the dog. Further, there is a shallow median fissure in the lower lip. A similar condition exists in the lips of all early marsupial embryos

I have been able to examine (*Macropus, Halmatura, Phalangista, Petrogale*).

It may be briefly stated that the fissured lip of the calf and lamb are the result of non-coalescence of the globular processes with the lateral bars which form the lips and maxilla. This, of course, applies to such cases in man. The fissure in the lip of the hare is due to the non-coalescence of the globular processes with each other, an event quite normal in this mammal, as in rodents generally; in the case of the pug, with median cleft in the lip and nose, the failure of union between these processes is a defect hereditarily transmitted.

Macrostoma is due to failure of union in the posterior part of the oral cleft, whilst the median cleft in the lower lip, an event of very rare occurrence, is due to some cause preventing the mandibular bar from fusing in the median line.—*Dental Record.*

